# NT-proBNP improves prediction of cardiorenal complications in type 2 diabetes: the Hong Kong Diabetes Biobank

**DOI:** 10.1007/s00125-024-06299-x

**Published:** 2024-11-07

**Authors:** Ronald C. W. Ma, Claudia H. T. Tam, Yong Hou, Eric S. H. Lau, Risa Ozaki, Juliana N. M. Lui, Elaine Chow, Alice P. S. Kong, Chuiguo Huang, Alex C. W. Ng, Erik G. Fung, Andrea O. Y. Luk, Wing Yee So, Cadmon K. P. Lim, Juliana C. N. Chan, for the Hong Kong Diabetes Biobank Study Group

**Affiliations:** 1 Department of Medicine and Therapeutics, The Chinese University of Hong Kong, Prince of Wales Hospital, Shatin, Hong Kong, HKSAR China; 2 Laboratory for Molecular Epidemiology in Diabetes, Li Ka Shing Institute of Health Sciences, The Chinese University of Hong Kong, Hong Kong, HKSAR China; 3 Chinese University of Hong Kong-Shanghai Jia Tong University Joint Research Centre in Diabetes Genomics and Precision Medicine, The Chinese University of Hong Kong, Hong Kong, HKSAR China; 4 Hong Kong Institute of Diabetes and Obesity, The Chinese University of Hong Kong, Hong Kong, HKSAR China; 5 Li Ka Shing Institute of Health Sciences, The Chinese University of Hong Kong, Hong Kong, HKSAR China

**Keywords:** Biomarkers, Cardiovascular, Complications, Epidemiology, Heart failure, Precision medicine

## Abstract

**Aims/hypothesis:**

N-terminal pro B-type natriuretic peptide (NT-proBNP) is a natriuretic peptide that is strongly associated with congestive heart failure (CHF). The utility of NT-proBNP for prediction of cardiovascular events and renal endpoints, compared with clinical risk factors, has not been evaluated in detail. We hypothesise that NT-proBNP can improve risk stratification and prediction of cardiorenal events in type 2 diabetes, beyond that provided by clinical risk factors.

**Methods:**

NT-proBNP was measured in 1993 samples from the Hong Kong Diabetes Biobank, a multicentre prospective diabetes cohort and biobank. A cut-off of ≥125 pg/ml was used to define elevated NT-proBNP. Associations between elevated NT-proBNP and incident cardiovascular and renal endpoints were examined using Cox regression, adjusted for sex, age and duration of diabetes, as well as other covariates. Prognostic and incremental predictive values of NT-proBNP in diabetes cardiorenal complications, compared with those of the Joint Asia Diabetes Evaluation risk equations for CHD, CHF and kidney failure, were evaluated using the concordance index (C index), net reclassification improvement index, integrated discrimination improvement index and relative integrated discrimination improvement index.

**Results:**

A total of 24.7% of participants had elevated NT-proBNP. Participants with elevated NT-proBNP at baseline had a more adverse cardiometabolic profile, with 2–4-fold higher frequency of complications at baseline. Adjusting for age at baseline, sex and duration of diabetes, elevated NT-proBNP was associated with incident atrial fibrillation (HR 4.64 [95% CI 2.44, 8.85]), CHD (HR 4.21 [2.46, 7.21]), CVD (HR 3.32 [2.20, 5.01]) and CHF (HR 4.18 [2.18, 8.03]; all *p*<0.001). All these associations remained significant after further adjustment for additional covariates. Elevated NT-proBNP had good discriminative ability for various cardiorenal endpoints, with C index of 0.83 (95% CI 0.76, 0.90) for CHD, 0.88 (0.81, 0.94) for atrial fibrillation, 0.89 (0.83, 0.95) for CHF, 0.81 (0.77, 0.84) for 40% drop in eGFR and 0.88 (0.84, 0.92) for kidney failure. Models incorporating NT-proBNP had improved prediction compared with established clinical risk models. Sensitivity analyses including alternative cut-off of NT-proBNP, as well as use of other risk engines of CHD, yielded similar results.

**Conclusions/interpretation:**

NT-proBNP demonstrated a promising ability to serve as a prognostic marker for a variety of cardiorenal complications in type 2 diabetes. Considering NT-proBNP in clinical assessments could potentially help identify high-risk individuals who may benefit from more intensive therapies.

**Graphical Abstract:**

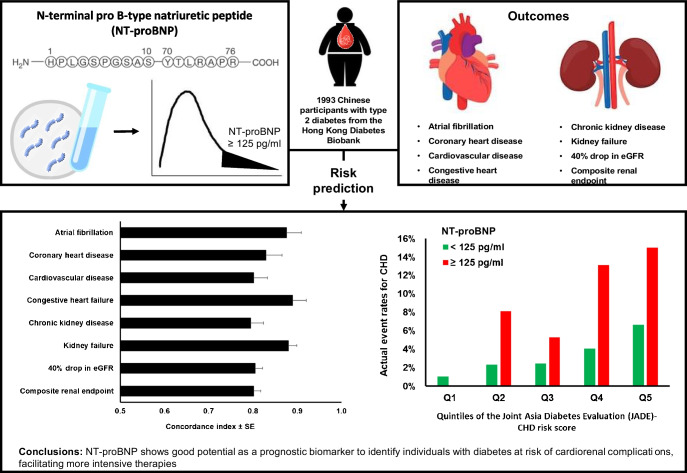

**Supplementary Information:**

The online version contains peer-reviewed but unedited supplementary material available at 10.1007/s00125-024-06299-x.


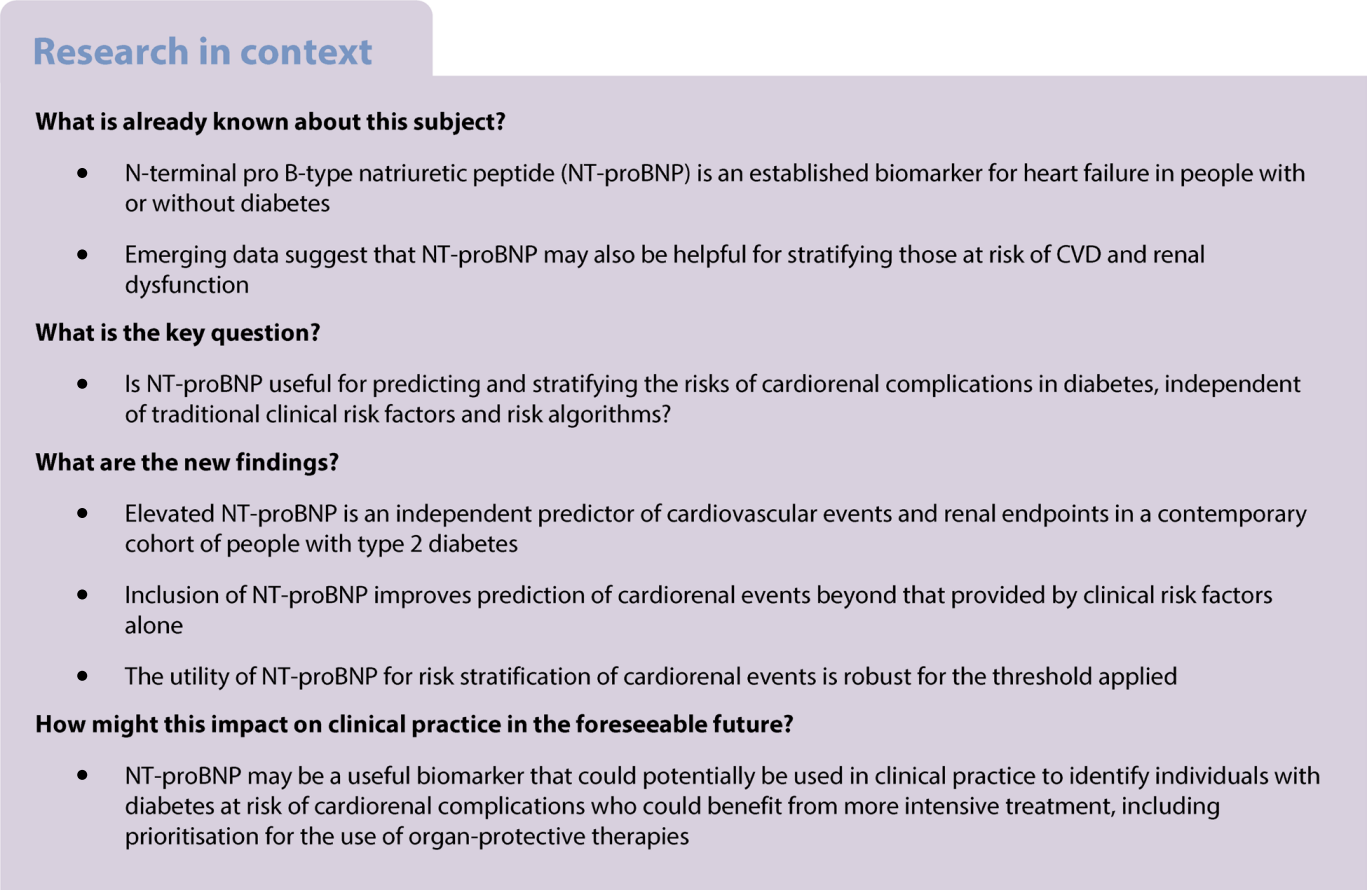


## Introduction

Recent availability of treatments that can reduce cardiovascular events and progression of diabetic kidney disease has heralded a new era of diabetes management, where there are exciting possibilities for modifying the trajectory towards significant complications and mortality [1, 2]. However, this has also highlighted the challenge in identifying individuals at risk of diabetes-related complications [3], in order to implement early intensive risk factor management, or to prioritise them for early initiation of organ-protective agents such as sodium–glucose cotransporter 2 inhibitor (SGLT2i) and glucagon-like peptide 1 receptor agonist (GLP-1RA) [4, 5]. Several approaches have been explored for the prediction of cardiovascular outcomes in clinical practice, and these fall under the category of precision prognostics in the current drive to implement precision medicine in diabetes [6, 7]. These approaches include using clinical risk equations such as the UK Prospective Diabetes Study (UKPDS) risk engine [8], using contemporary risk models such as the Risk Equations for Complications Of type 2 Diabetes (RECODe) equations [3], use of biomarkers including genome-wide polygenic risk score [9–11], as well as use of traditional biomarkers such as microalbuminuria [12]. As highlighted in a recent consensus report on precision medicine in diabetes, previous studies investigating these different strategies of patient risk stratifications often had different limitations, in particular insufficient validation studies, lack of standardisation of the biomarker measurement, limited access and availability, and inadequate comparison with current practice [3, 7].

N-terminal pro B-type natriuretic peptide (NT-proBNP) is a cleaved inactive peptide of B-type natriuretic peptide produced from the myocardium in response to cardiac ventricular stress, and has been shown to be a reliable biomarker for heart failure and other cardiovascular complications in individuals with or without diabetes [13, 14]. It has also been proposed as a biomarker for selecting high-risk individuals for participation in clinical trials of heart failure [15]. Recent data suggest it may also be a useful biomarker for renal dysfunction [16]. Given limited data from Asian populations on use of the biomarker, or comprehensive evaluation of the utility of NT-proBNP for various cardiorenal complications in diabetes, we undertook this study to evaluate the clinical utility of NT-proBNP for predicting cardiorenal complications in a longitudinal cohort of Chinese individuals with type 2 diabetes, including comparison of its performance in precision prognostics compared with established clinical risk factors.

## Methods

### Study population

All participants included in the current analysis were from the Hong Kong Diabetes Biobank (HKDB). The HKDB is a multicentre prospective cohort study co-ordinated by the Chinese University of Hong Kong (NCT05282680). The HKDB was set up using a similar design to that of the Hong Kong Diabetes Register (HKDR), established at the Prince of Wales Hospital (PWH) and the Chinese University of Hong Kong in 1995, with regular comprehensive assessment of diabetes-related risk factors and also comprehensive screening of diabetes complications, as previously described [17]. In 2000, the Hong Kong Hospital Authority (HA) set up a territory-wide diabetes risk assessment programme via establishing hospital-based diabetes centres and adopting a similar assessment method for diabetes complications [17]. Initiated in 2014, the HKDB enrolled participants from 11 diabetes centres at major public hospitals across Hong Kong using similar enrolment and assessment methods, aiming to establish a multicentre diabetes register and biobank for biomarker discovery using a transomics approach [12], as well as a platform to validate and compare known biomarkers.

**Fig. 1:**
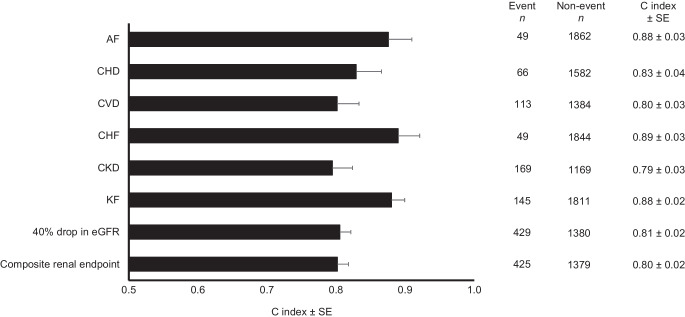
C index for predicting incident diabetes cardiorenal complications using only the binary NT-proBNP values (≥125 vs <125). 'Composite renal endpoint' was defined as either 40% drop in eGFR or KF. The prediction model includes NT-proBNP only. The C index and SE were estimated based on the risk prediction models computed by Cox regression

The design of the study, recruitment methods, baseline data collection and biochemical investigations have been described in detail before [12, 18, 19]. Briefly, the HKDB was set up following protocols similar to those used for the HKDR. All individuals were invited for participation and prospective follow-up at the time of their regular structured assessment of risk factors, biochemical control and presence of diabetes complications, based on the modified European DIABCARE protocol [20]. Once enrolled, participants were prospectively followed until their death. Over the period from 2014 to 2019, the study consecutively enrolled more than 20,000 individuals with diabetes. From this total participant population, baseline NT-proBNP levels were measured in a subset of 2000 individuals. Ultimately, 1993 eligible participants with median follow-up duration of 5.1 years were included in the analysis. The derivation of the eligible analysis cohort is depicted in electronic supplementary material (ESM) Fig. 1.

While the hospitalised setting of the HKDB study may lead to a bias towards more severe or complex diabetes cases, the large and diverse sample collected from various major public hospitals across different geographical areas of Hong Kong still reflects a significant portion of the population with diabetes in the region. Therefore, we compared the baseline clinical characteristics of participants included in this study with those who were not (ESM Table 1). Our results indicate no notable differences in the baseline clinical characteristics between the two groups of participants. At enrolment, participants consented for prospective follow-up and biobanking of blood samples for research. All participants provided written informed consent and the study was approved by the Joint Chinese University of Hong Kong–New Territories East Cluster Clinical Research Ethics Committee and the Clinical Research Ethics Committee of each participating hospital [12].

### Demographic and laboratory measurements

Demographic information, medical history and medications were collected from each participant via face-to-face interviews with standardised questionnaires. Data on race/ethnicity and sex were abstracted from medical records. Blood pressure was measured in both arms after sitting for ≥5 min and the mean value was used for analysis. Anthropometric measurements were taken for BMI calculation. Blood samples after at least 8 h of overnight fasting were measured for HbA_1c_, serum creatinine and lipid profile with certificated routine assays at local laboratories. Albumin was quantified in a random spot urine sample using immunoturbidimetry [12]. Serum creatinine was measured by Jaffe’s kinetic method [12] and eGFR was calculated using the Chronic Kidney Disease Epidemiology Collaboration (CKD-EPI) equation [21].

### Measurements of NT-proBNP

All blood samples for biobanking were transported back to the PWH in cooled and temperature-monitored containers and processed within 4 h. All samples were centrally stored at −80°C at the PWH in secured freezers with remote temperature monitoring. All samples used for the current measurement of NT-proBNP were free from any previous freeze–thaw cycles. NT-proBNP was measured in archived samples using an electrochemiluminescent immunoassay on a Roche Cobas e401 analyser (Roche Diagnostics, Switzerland).

### Clinical outcomes

In the HKDB studies, the CHD endpoint was defined using the International Classification of Diseases, Ninth Revision. Ascertainment of CHD was based on a composite of acute myocardial infarction, nonfatal ischaemic heart disease or angina pectoris. For the analysis of prevalent complications, participants with prevalent CHD or cardiovascular complications previously diagnosed prior to the recruitment visit were classified as cases. CVD was defined as the occurrence of CHD, stroke (defined as the occurrence of ischaemic stroke except transient ischaemic attack, haemorrhagic stroke or acute but ill-defined cerebrovascular disease) or peripheral vascular disease (PVD, defined as the occurrence of amputation, gangrene or peripheral revascularisation). Congestive heart failure (CHF) was defined as hospitalisation for CHF. Any type of atrial fibrillation (AF) was defined as according to clinical diagnosis of AF based on 12-lead ECGs. Chronic kidney disease (CKD) was defined as eGFR<60 ml/min per 1.73 m^2^ according to the CKD-EPI equation. Kidney failure (KF) was defined as eGFR<15 ml/min per 1.73 m^2^, initiation of dialysis or renal transplantation. The surrogate renal endpoint of 40% drop in eGFR was defined as a drop in eGFR of 40% or more during the follow-up period. The combined renal endpoint was defined as onset of KF or drop in eGFR of 40% or more during follow-up.

For the prospective analysis, we excluded prevalent cases and examined only incident events during follow-up. Follow-up time was calculated as the period from enrolment to the first occurrence of endpoint, the date of death or 31 December 2019, whichever came first. Incident cardiovascular complications were defined as new-onset cardiovascular endpoints that occurred during the follow-up period.

### Statistical analyses

All analyses were performed using SPSS for Windows v26 (SPSS, IBM, Chicago, IL, USA) and R v3.4.4 (http://www.r-project.org/, 31 December 2019). To improve the validity of the multiple regression models, skewed quantitative traits were transformed to approximate a normal distribution using the natural logarithm function. Data are described with percentage (*n*), mean±SD or median (Q1–Q3). Differences between groups were tested with *χ*^2^ test, Student’s *t* test or Mann–Whitney test, as appropriate. A two-tailed *p* value <0.05 was considered statistically significant.

This study did not employ any imputation techniques to handle missing data, and all analyses were conducted using the available, non-imputed data. Individuals with missing data points for any variables included in the logistic or linear regression model were removed from the analysis.

To study the clinical importance of high NT-proBNP levels, individuals were categorised into two groups using the established threshold of 125 pg/ml (i.e. high NT-proBNP was defined as ≥125 pg/ml) [22, 23]. As a sensitivity analysis, we also applied an alternative threshold of ≥400 pg/ml as the definition of elevated NT-proBNP, as suggested by some studies conducted among Asian populations [24].

In the cross-sectional analysis, we examined the association between high NT-proBNP and prevalent diabetes cardiorenal complications using logistic regression with adjustment for sex, age and duration of diabetes. ORs with their 95% CIs were calculated. In the prospective analysis, associations of high NT-proBNP with incident cardiovascular and renal outcomes were assessed by Cox regression, adjusted for sex, age and duration of diabetes. HRs with the corresponding 95% CIs were calculated. We further tested for the independent effect of NT-proBNP levels by adjusting for different baseline covariates, including smoking status, BMI, waist circumference (WC), systolic and diastolic blood pressure (SBP and DBP), HbA_1c_, triacylglycerol (TG), HDL-cholesterol, LDL-cholesterol, albumin/creatinine ratio (ACR), eGFR and use of medications. To account for the multiple comparisons, we applied the Bonferroni correction when analysing the three adjustment models for eight outcome variables of interest (*p* value threshold=0.05/(3×8)=2.1×10^−3^).

The concordance index (C index), net reclassification improvement (NRI) index, integrated discrimination improvement (IDI) index and relative IDI index were used to assess the prognostic and incremental predictive values of NT-proBNP in diabetes cardiorenal complications, over the Joint Asia Diabetes Evaluation (JADE) risk equations for CHD, CHF and KF, which were developed in Chinese individuals with type 2 diabetes [25–28]. The calculations of these indices were based on the predicted risk computed by the Cox regression model, using the ‘concordance.index’, ‘nricens’ and ‘IDI.INF’ functions in the R package. The 95% CIs for all indices were estimated using the bootstrap resampling method with 10,000 iterations. We compared two correlated C indices using the paired *t* test implemented by the ‘cindex.comp’ function in the R package.

## Results

### Baseline characteristics of participants

A total of 1993 individuals with type 2 diabetes were included in the analysis. The baseline characteristics are shown in Table 1. A total of 24.7% of participants had elevated NT-proBNP. When stratified according to NT-proBNP of <125 pg/ml or ≥125 pg/ml, participants with elevated NT-proBNP were older, were more often male, had a longer duration of diabetes, had a higher WC among female participants, had a higher WHR and had a more adverse lipid profile, with higher LDL-cholesterol, lower HDL-cholesterol, higher SBP, higher urine ACR and lower eGFR. They were also more likely to be receiving lipid-lowering medications, anti-hypertensive medications and oral glucose-lowering drugs, as well as insulin.

**Table 1:** Clinical characteristics for all participants and stratified by the cut-off of NT-proBNP in the HKDB study

Characteristic	ALL (*N*=1993)	NT-proBNP<125 (*n*=1501)	NT-proBNP≥125 (*n*=492)	*p*
Clinical characteristics at baseline				
Male	59.8 (1192)	61.2 (919)	55.5 (273)	0.0243
Age (years)	61.1±11.0	58.8±10.4	68.0±9.69	<0.0001
Age at onset (years)	49.7±11.6	48.6±11.1	53.1±12.3	<0.0001
Duration of diabetes (years)	11.3±8.66	10.2±8.18	14.6±9.22	<0.0001
Smoking status				0.1799
Non-smoker	66.1 (1317)	67.2 (1008)	62.8 (309)	
Ex-smoker	22.0 (438)	20.9 (313)	25.4 (125)	
Current smoker	11.9 (237)	11.9 (179)	11.8 (58)	
Body height (m)	1.62±0.08	1.63±0.08	1.60±0.08	<0.0001
Body weight (kg)	68.4 (60.0–78.0)	68.9 (60.4–78.4)	67.6 (59.2–76.5)	0.0377
BMI (kg/m^2^)	25.9 (23.4–29.1)	25.8 (23.3–28.9)	26.2 (23.7–29.5)	0.1082
WC (cm)				
Men	93.9±11.3	93.6±11.0	95.0±12.0	0.0745
Women	89.5±11.4	88.7±11.0	91.7±12.2	0.0009
Hip circumference (cm)	94.7±8.5	94.8±8.3	94.4±9.2	0.3600
WHR	0.97±0.08	0.96±0.07	0.99±0.08	<0.0001
HbA_1c_ (mmol/mol)	55.0 (49.0–65.0)	55.0 (48.0–65.0)	56.0 (50.0–66.0)	0.0544
HbA_1c_ (%)	7.20 (6.60–8.10)	7.20 (6.50–8.10)	7.30 (6.68–8.20)	0.0544
Total cholesterol (mmol/l)	4.26 (3.73–4.86)	4.29 (3.79–4.88)	4.16 (3.60–4.76)	0.0021
TGs (mmol/l)	1.34 (0.96–2.00)	1.34 (0.95–1.98)	1.35 (0.99–2.07)	0.3463
HDL-cholesterol (mmol/l)	1.22 (1.01–1.47)	1.23 (1.01–1.48)	1.20 (0.98–1.46)	0.0485
LDL-cholesterol (mmol/l)	2.26 (1.81–2.75)	2.29 (1.83–2.76)	2.14 (1.72–2.71)	0.0069
SBP (mmHg)	135±18.4	133±16.8	144±20.1	<0.0001
DBP (mmHg)	74.0±11.4	74.1±11.0	73.5±12.4	0.3346
ACR	3.90 (1.10–24.6)	2.50 (0.90–10.6)	26.5 (3.90–152)	<0.0001
eGFR (min/ml per 1.73 m^2^)	75.9±26.3	83.3±21.9	53.5±26.0	<0.0001
Treatment at baseline				
Lipid-lowering drug	68.7 (1359)	66.6 (993)	75.0 (366)	0.0006
Blood pressure anti-hypertensive	77.2 (1523)	71.8 (1068)	93.4 (455)	<0.0001
Oral glucose-lowering drug	86.0 (1682)	89.3 (1319)	75.8 (363)	<0.0001
Insulin treatment	38.1 (749)	33.8 (501)	51.0 (248)	<0.0001
History of cardiorenal complication at baseline				
AF	2.6 (52)	0.4 (6)	9.8 (46)	<0.0001
CHD	17.3 (345)	12.1 (181)	33.3 (164)	<0.0001
Stroke	8.8 (176)	6.5 (98)	15.9 (78)	<0.0001
PVD	1.6 (32)	0.7 (11)	4.3 (21)	<0.0001
CVDs	24.9 (496)	18.1 (271)	45.7 (225)	<0.0001
CHF	5.0 (100)	1.4 (21)	16.1 (79)	<0.0001
CKD	32.9 (655)	21.5 (322)	67.7 (333)	<0.0001
KF	1.9 (37)	0.3 (5)	6.5 (32)	<0.0001
40% drop in eGFR	9.1 (181)	5.9 (89)	18.7 (92)	<0.0001
Composite renal endpoint	9.3 (186)	6 (90)	19.5 (96)	<0.0001
Incident cardiorenal complication during follow-up (without history)				
AF	2.6 (49)	1.1 (17)	7.6 (32)	<0.0001
CHD	4.0 (66)	2.4 (32)	10.4 (34)	<0.0001
Stroke	3.1 (56)	2.4 (33)	5.6 (23)	0.0009
PVD	1.5 (29)	0.8 (12)	3.6 (17)	<0.0001
CVDs	7.5 (113)	5.2 (64)	18.4 (49)	<0.0001
CHF	2.6 (49)	1.1 (16)	8.0 (33)	<0.0001
CKD	12.6 (169)	10.1 (119)	31.4 (50)	<0.0001
KF	7.4 (145)	3.3 (50)	20.7 (95)	<0.0001
40% drop in eGFR	23.7 (429)	16.3 (229)	50 (200)	<0.0001
Composite renal endpoint	23.6 (425)	16.3 (229)	49.5 (196)	<0.0001

Data are expressed as percentage (*n*), mean±SD or median (Q1–Q3)

Between-group comparisons were performed by *χ*^2^ test for categorical variables, and unpaired Student’s *t* test or the Wilcoxon rank sum test for continuous variables

The percentages in this table are based on available data. Discrepancies may arise due to missing data

### Association between NT-proBNP and prevalent diabetes cardiorenal complications

Comparing the prevalence of diabetes-related vascular complications at baseline, participants with elevated NT-proBNP had a frequency of complications at baseline 2–4 times higher compared with those with NT-proBNP<125 pg/ml (Table 1). Using logistic regression, adjusting for sex, age and diabetes duration, elevated NT-proBNP≥125 pg/ml was significantly associated with an increased risk of AF, CHD, CVD and CKD (Model 1 in Table 2). Analysis with NT-proBNP as a continuous trait yielded similar results (ESM Table 2).

**Table 2:** Association of binary NT-proBNP (≥125 vs <125) with prevalent and incident diabetes cardiorenal complications

Outcome	Adjustments	Prevalent outcomes				Incident outcomes			
		*n*		Logistic regression		*n*		Cox regression	
		Case	Control	OR (95% CI)	*p*	Event	Non-event	HR (95% CI)	*p*
AF	Model 1: Sex, age, duration of diabetes	52	1901	21.3 (8.63, 52.5)	3.2×10^−11^	49	1852	4.64 (2.44, 8.85)	3.0×10^−6^
	Model 2: Model 1, smoking status, lnBMI, WC, SBP, DBP, lnHbA_1c_, lnTG, lnHDL-C, lnLDL-C, use of BP drugs, use of oral glucose-lowering drug, use of insulin, use of LLD	49	1798	23.0 (8.89, 59.4)	9.8×10^−11^	46	1752	4.65 (2.29, 9.45)	2.1×10^−5^
	Model 3: Model 2, lnACR, eGFR	46	1528	31.0 (11.0, 88.0)	1.0×10^−10^	44	1484	3.60 (1.67, 7.72)	1.0×10^−3^
CHD	Model 1: Sex, age, duration of diabetes	345	1638	2.73 (2.08, 3.58)	3.4×10^−13^	65	1572	4.21 (2.46, 7.21)	1.6×10^−7^
	Model 2: Model 1, smoking status, lnBMI, WC, SBP, DBP, lnHbA_1c_, lnTG, lnHDL-C, lnLDL-C, use of BP drugs, use of oral glucose-lowering drug, use of insulin, use of LLD	319	1552	2.35 (1.72, 3.20)	8.2×10^−8^	59	1492	3.80 (2.07, 6.97)	1.7×10^−5^
	Model 3: Model 2, lnACR, eGFR	287	1311	2.55 (1.78, 3.63)	2.5×10^−7^	57	1253	2.84 (1.47, 5.48)	1.9×10^−3^
CVD	Model 1: Sex, age, duration of diabetes	496	1487	2.74 (2.14, 3.50)	8.3×10^−16^	111	1376	3.32 (2.20, 5.01)	1.1×10^−8^
	Model 2: Model 1, smoking status, lnBMI, WC, SBP, DBP, lnHbA_1c_, lnTG, lnHDL-C, lnLDL-C, use of BP drugs, use of oral glucose-lowering drug, use of insulin, use of LLD	463	1408	2.38 (1.78, 3.16)	3.3×10^−9^	102	1306	2.96 (1.87, 4.69)	3.5×10^−6^
	Model 3: Model 2, lnACR, eGFR	414	1184	2.60 (1.87, 3.60)	1.1×10^−8^	96	1088	2.35 (1.42, 3.89)	9.0×10^−4^
CHF	Model 1: Sex, age, duration of diabetes	99	1884	11.6 (6.78, 20.0)	5.1×10^−19^	49	1835	4.18 (2.18, 8.03)	1.8×10^−5^
	Model 2: Model 1, smoking status, lnBMI, WC, SBP, DBP, lnHbA_1c_, lnTG, lnHDL-C, lnLDL-C, use of BP drugs, use of oral glucose-lowering drug, use of insulin, use of LLD	92	1779	8.49 (4.80, 15.0)	1.9×10^−13^	48	1731	3.84 (1.93, 7.63)	1.2×10^−4^
	Model 3: Model 2, lnACR, eGFR	85	1513	8.30 (4.38, 15.7)	9.2×10^−11^	45	1468	2.61 (1.27, 5.37)	9.4×10^−3^
CKD	Model 1: Sex, age, duration of diabetes	652	1331	4.80 (3.73, 6.16)	1.6×10^−34^	168	1163	2.51 (1.76, 3.57)	3.3×10^−7^
	Model 2: Model 1, smoking status, lnBMI, WC, SBP, DBP, lnHbA_1c_, lnTG, lnHDL-C, lnLDL-C, use of BP drugs, use of oral glucose-lowering drug, use of insulin, use of LLD	616	1255	3.94 (2.96, 5.24)	3.7×10^−21^	159	1096	2.12 (1.46, 3.09)	8.5×10^−5^
	Model 3: Model 2, lnACR, eGFR	579	1019	0.84 (0.49, 1.46)	0.5424	147	872	1.34 (0.89, 2.02)	0.1572
KF	Model 1: Sex, age, duration of diabetes	37	1946	19.6 (7.18, 53.3)	6.2×10^−9^	144	1802	5.99 (4.11, 8.72)	1.0×10^−20^
	Model 2: Model 1, smoking status, lnBMI, WC, SBP, DBP, lnHbA_1c_, lnTG, lnHDL-C, lnLDL-C, use of BP drugs, use of oral glucose-lowering drug, use of insulin, use of LLD	34	1837	10.7 (3.55, 32.2)	2.6×10^−5^	128	1709	3.70 (2.43, 5.64)	1.1×10^−9^
	Model 3: Model 2, lnACR, eGFR	33	1565	2.02 (0.50, 8.17)	0.3227	123	1442	1.90 (1.20, 2.99)	5.7×10^−3^
40% drop in eGFR	Model 1: Sex, age, duration of diabetes	181	1799	3.22 (2.28, 4.55)	2.8×10^−11^	426	1373	3.13 (2.53, 3.87)	3.5×10^−26^
	Model 2: Model 1, smoking status, lnBMI, WC, SBP, DBP, lnHbA_1c_, lnTG, lnHDL-C, lnLDL-C, use of BP drugs, use of oral glucose-lowering drug, use of insulin, use of LLD	173	1697	2.51 (1.71, 3.69)	2.6×10^−6^	395	1302	2.46 (1.96, 3.09)	9.0×10^−15^
	Model 3: Model 2, lnACR, eGFR	158	1439	2.02 (1.31, 3.12)	1.4×10^−3^	364	1075	1.47 (1.15, 1.86)	1.8×10^−3^
Composite renal endpoint	Model 1: Sex, age, duration of diabetes	186	1794	3.37 (2.40, 4.74)	2.7×10^−12^	422	1372	3.06 (2.47, 3.78)	5.6×10^−25^
	Model 2: Model 1, smoking status, lnBMI, WC, SBP, DBP, lnHbA_1c_, lnTG, lnHDL-C, lnLDL-C, use of BP drugs, use of oral glucose-lowering drug, use of insulin, use of LLD	176	1694	2.55 (1.74, 3.74)	1.5×10^−6^	393	1301	2.42 (1.93, 3.04)	2.8×10^−14^
	Model 3: Model 2, lnACR, eGFR	161	1436	2.03 (1.32, 3.11)	1.3×10^−3^	362	1074	1.45 (1.14, 1.84)	2.6×10^−3^

ORs and 95% CIs, as well as HRs and 95% CIs, are reported according to high NT-proBNP levels

*p* was obtained from logistic or Cox regression models with adjustments for covariates

LLD, lipid-lowering drugs; lnACR, log_*e*_-transformation of ACR; lnBMI, log_*e*_-transformation of BMI; lnHbA_1c_, log_*e*_-transformation of HbA_1c_; lnHDL-C, log_*e*_-transformation of HDL-cholesterol; lnLDL-C, log_*e*_-transformation of LDL-cholesterol; lnTG, log_*e*_-transformation of TG levels

In multivariate logistic regression adjusting for covariates at baseline, including sex, age, diabetes duration, smoking status, BMI, WC, SBP, DBP, HbA_1c_, TG, HDL-cholesterol, LDL-cholesterol and use of medications, elevated NT-proBNP≥125 pg/ml was associated with significantly increased risk of AF (OR 23.0 [95% CI 8.89, 59.4]), CHD (OR 2.35 [1.72, 3.20]), CVD (OR 2.38 [1.78, 3.16]) and CHF (OR 8.49 [4.80, 15.0]) (Model 2 in Table 2). All these associations remained significant after further adjustment for renal dysfunction with inclusion of baseline ACR and eGFR, some with numerically higher OR (Model 3 in Table 2). Elevated NT-proBNP≥125 pg/ml was also associated with increased risk of CKD (OR 3.94 [2.96, 5.24]) and KF (OR 10.7 [3.55, 32.2]) after adjustment for the baseline covariates in Model 2 (Table 2). Further adjustment for baseline ACR and eGFR diminished the association with renal outcomes (Model 3 in Table 2). The associations for all cardiorenal outcomes held true even when we corrected for multiple comparisons.

### NT-proBNP and incident cardiovascular complications

As shown in Table 1, participants with NT-proBNP≥125 pg/ml experienced a greater number of incident cardiorenal complications, with 2–6 times higher frequency, over the median 5.1 years of follow-up compared with those with NT-proBNP<125 pg/ml. In analyses adjusting for sex, age and duration of diabetes, elevated NT-proBNP≥125 pg/ml was associated with incident AF (HR 4.64 [95% CI 2.44, 8.85]), CHD (HR 4.21 [2.46, 7.21]), CVD (HR 3.32 [2.20, 5.01]) and CHF (HR 4.18 [2.18, 8.03]) (Model 1 in Table 2). All these associations remained significant after further adjustments for smoking status, BMI, WC, SBP, DBP, HbA_1c_, TG, HDL-cholesterol, LDL-cholesterol, use of medications, ACR and eGFR at baseline (Model 3 in Table 2). The results remain significant after Bonferroni adjustment, except for CHF with the Model 3 adjustments.

### NT-proBNP and incident renal complications

Elevated NT-proBNP≥125 pg/ml was associated with incident CKD (HR 2.51 [1.76, 3.57]), a 40% decrease in eGFR during follow-up (HR 3.13 [2.53, 3.87]) and KF (HR 5.99 [4.11, 8.72]). All these associations remained significant after further adjustments for baseline covariates in Model 2 (Table 2). Further adjustment for baseline ACR and eGFR rendered the association with incident CKD non-significant (HR 1.34 [0.89, 2.02]), although the associations with 40% decrease in eGFR (HR 1.47 [1.15, 1.86]), KF (HR 1.90 [1.20, 2.99]), or a composite renal endpoint of 40% drop in eGFR or KF (HR 1.45 [1.14, 1.84]), remained significant despite reduction in the strength of the association (Model 3 in Table 2).

### Clinical utility of NT-proBNP for predicting cardiorenal endpoints in diabetes

The utility of NT-proBNP for predicting different cardiorenal endpoints in diabetes was next examined. Elevated NT-proBNP was found to have good discrimination ability to identify those at risk of future endpoints, with C index that ranged from 0.80 (95% CI 0.74, 0.86) for CVD, to 0.83 (0.76, 0.90) for CHD, to 0.88 (0.81, 0.94) for AF, and was highest for CHF at 0.89 (0.83, 0.95) (Fig. 1). When compared with established risk prediction equations for CHD, CHF and KF, incorporating NT-proBNP enhanced prediction of each complication beyond that provided by clinical risk factors alone, with significant incremental increase in C index (Table 3). Likewise, analysis using NRI indicated a significant contribution of adding NT-proBNP to reclassification (Table 4). IDI and/or relative IDI were significant for incident CHD and CHF (Table 4).

**Table 3:** C index of NT-proBNP in predicting incident cardiorenal complications, over the established clinical risk scores

Outcome	Clinical risk score [Ref]	Predictors included in the clinical risk score	*n*		ROC curve analysis				
			Case	Control	Model 1: NT-proBNP only C index (95% CI)	Model 2 (base model): Clinical risk score only C index (95% CI)	Model 3: NT-proBNP and clinical risk score C index (95% CI)	Comparison of C index Model 3 vs Model 2	
								Increase in C index	*p*_increase_
CHD	JADE-CHD risk equation [26]	Age, sex, current smoking status, duration of diabetes, eGFR, urinary ACR and non-HDL-cholesterol	62	1311	0.82 (0.74, 0.89)	0.73 (0.66, 0.79)	0.75 (0.68, 0.81)	0.0210	0.0416
CHF	JADE-CHF risk equation [46]	Age, BMI, HbA_1c_, urinary ACR, blood haemoglobin and CHD during follow-up	45	1465	0.87 (0.80, 0.95)	0.80 (0.75, 0.86)	0.81 (0.75, 0.87)	0.0090	1.1×10^−3^
KF	JADE-KF risk equation [27]	eGFR, haematocrit, urinary ACR	132	1436	0.86 (0.82, 0.91)	0.94 (0.92, 0.96)	0.94 (0.92, 0.96)	0.0002	3.2×10^−3^

NT-proBNP was used as a binary variable with a predefined threshold of 125 pg/ml (i.e. high NT-proBNP was defined as ≥125 pg/ml)

For the C index analysis, Model 1 includes the NT-proBNP only. Model 2 (base model) includes the clinical risk score only. Model 3 includes both Models 1 and 2. C index±SE and related changes were estimated based on the risk prediction models (i.e. Models 1–3) computed by Cox regression

*p* was obtained from the test with the null hypothesis that 'C index is equal to zero'. *p*_increase_ was obtained from the test with the null hypothesis that 'Difference in C index between Models 2 and 3 is equal to zero'

While each analysis in Fig. 1 encompassed all participants with complete data on NT-proBNP and outcome, the analysis in Table 3 also incorporated clinical data. This resulted in a smaller sample size due to incomplete data on clinical variables

[Ref], reference citation

**Table 4:** NRI, IDI and relative IDI of NT-proBNP in predicting incident cardiorenal complications, over the JADE risk equations

Outcome	Clinical risk score [Ref]	Predictors included in the clinical risk score	*n*		NRI index		IDI index		Relative IDI index	
			Case	Control	Continuous NRI (95% CI)	*p*_NRI_	IDI (95% CI)	*p*_IDI_	rIDI (95% CI)	*p*_rIDI_
CHD	JADE-CHD risk equation [26]	Age, sex, current smoking status, duration of diabetes, eGFR, urinary ACR and non-HDL-cholesterol	62	1311	69.0% (43.0%, 94.4%)	<0.05	0.014 (0.003, 0.038)	1.0×10^−3^	0.490 (0.045, 0.934)	<0.05
CHF	JADE-CHF risk equation [46]	Age, BMI, HbA_1c_, urinary ACR, blood haemoglobin and CHD during follow-up	45	1465	90.3% (60.5%, 100%)	<0.05	0.008 (0.001, 0.029)	0.0250	0.093 (−0.127, 0.313)	>0.05
KF	JADE-KF risk equation [27]	eGFR, haematocrit, urinary ACR	132	1436	87.7% (70.4%, 100%)	<0.05	0.000 (−0.003, 0.008)	1.0000	0.005 (−0.024, 0.035)	>0.05

NT-proBNP was used as a binary variable with a predefined threshold of 125 pg/ml (i.e. high NT-proBNP was defined as ≥125 pg/ml)

For the NRI and IDI analyses, the old model includes the clinical risk score. The new model includes the old model and NT-proBNP

*p*_NRI_ was obtained from the test with the null hypothesis that 'Continuous NRI is equal to zero'. *p*_IDI_ was obtained from the test with the null hypothesis that 'IDI is equal to zero'. *p*_rIDI_ was obtained from the test with the null hypothesis that 'Relative IDI is equal to zero'

[Ref], reference citation; rIDI, relative IDI

Elevated NT-proBNP had C index of 0.79 (0.74, 0.85) for incident CKD, 0.81 (0.77, 0.84) for 40% drop in eGFR and 0.88 (0.84, 0.92) for KF (Fig. 1). Compared with an established risk equation for KF generated in a similar population [27], a model combining the risk equation and adding NT-proBNP achieved a C index of 0.94 (0.92, 0.96), with significant increase in C index compared with prediction of clinical risk score alone (Table 3). Analysis using NRI indicated improved prediction of KF after incorporating NT-proBNP, although there was no improvement using either IDI or relative IDI (Table 4).

The actual event rates for incident CHD, CHF and KF, stratified by the quintiles of JADE risk score and NT-proBNP, are shown in ESM Fig. 2 and ESM Table 3. Irrespective of the JADE risk score quintile, the event rate for each outcome was generally higher in participants with NT-proBNP≥125 pg/ml compared with those with NT-proBNP<125 pg/ml.

### Sensitivity analyses

Analyses between NT-proBNP and incident complications were repeated using log NT-proBNP as a continuous trait, as well as comparisons of top quintiles of NT-proBNP vs other quintiles, which all yielded consistent and similar significant associations, with numerically higher HR for most complications in comparisons of top quintiles compared with other quintiles (ESM Table 2).

A different cut-off of NT-proBNP of ≥400 pg/ml, which has been adopted in some Asian countries such as Japan, was also examined. A total of 183 participants (9.2%) had NT-proBNP of ≥400 pg/ml. Analysis using this cut-off yielded very similar results to the primary analysis, with significant association with incident CHD, CVD, CHF, as well as CKD and KF, with mostly similar effect size compared with stratification using the cut-off of 125 pg/ml (ESM Table 2).

While it is recommended to compare biomarkers against risk equations most appropriate to the population being studied [3, 7], we have also explored the added benefit of incorporating NT-proBNP, compared with other risk prediction equations developed in non-Asian populations. We used the updated UKPDS risk engine for ischaemic heart disease outcome [29], as well as the RECODe risk equation for myocardial infarction [30]. These were developed in individuals with type 2 diabetes in the UK and USA, respectively. The results were similar to the analysis using the JADE risk equations, whereby incorporation of NT-proBNP improved the prediction of cardiovascular events compared with using the clinical risk factors alone (ESM Table 4).

## Discussion

In the well-characterised HKDB, NT-proBNP was measured in 1993 participants and our main findings include: (1) elevated NT-proBNP, using the established cut-off of 125 pg/ml, was associated with increased risk of cardiovascular complications including AF, CHD, CHF and CVD; (2) elevated NT-proBNP was associated with increased risk of CKD and KF; (3) elevated NT-proBNP, independent of clinical risk factors, was associated with different incident cardiovascular complications; (4) elevated NT-proBNP, independent of clinical risk factors, was associated with incident renal dysfunction including CKD, 40% decline in eGFR and KF; (5) NT-proBNP alone shows good discriminating ability for identifying people with type 2 diabetes for cardiorenal complications, and improves upon prediction using established risk equations for cardiorenal complications. Our results highlight the potential utility of incorporating NT-proBNP as a single biomarker to identify individuals with type 2 diabetes who are at risk of cardiorenal endpoints, and highlight its utility in patient stratification and potential prioritisation for use of organ-protective agents with cardiorenal benefits.

### NT-proBNP and CHF

A large body of evidence supports the use of NT-proBNP in risk stratification and prognostication for heart failure, and this has been incorporated into major clinical management guidelines in many parts of the world. For example, in the European Society of Cardiology guidelines for the diagnosis and treatment of acute and chronic heart failure, measurements of natriuretic peptides such as NT-proBNP are recommended for the diagnosis and prognostication of heart failure, and levels below 125 pg/ml make a diagnosis of heart failure unlikely [23, 31, 32]. This aligns with the NT-proBNP thresholds observed to be predictive of CHF outcomes in the current study. The utility of NT-proBNP for predicting heart failure has also been consistently demonstrated among individuals with type 2 diabetes. In the CANagliflozin cardioVascular Assessment Study (CANVAS) trial, which recruited individuals with type 2 diabetes at high cardiovascular risk, measurement of NT-proBNP at baseline, 1 year and 6 years found that elevated NT-proBNP of >125 pg/ml at baseline was prognostic of incident hospitalisation with heart failure (HR 5.40 [95% CI 2.67, 10.9]) [33], with an effect size similar to that observed in our current analysis in the HKDB (HR 4.18 [2.18, 8.03]). Furthermore, it was noted that NT-proBNP levels were lower at year 1 and year 6 in the canagliflozin arm compared with the placebo arm, highlighting the effects of SGLT2i in lowering NT-proBNP [33]. Interestingly, mediation analysis in the trial suggested that 10.4% of the effects of canagliflozin on hospitalisation with heart failure were reflected in the lowering of NT-proBNP, suggesting that NT-proBNP is one of the mechanistic links between SGLT2i and its cardioprotective effects.

### NT-proBNP and cardiovascular complications

Several previous studies have explored the role of NT-proBNP as a biomarker for cardiovascular risk, providing important context for the findings from our current analysis. In a meta-analysis of 40 prospective studies, NT-proBNP was found to facilitate prediction of CHD and stroke [34], consistent with the cardiovascular risk associations observed in the HKDB. In a prospective study of 631 individuals with diabetes, it was noted that elevated NT-proBNP of >125 pg/ml had an area under receiver operating characteristic (ROC) curve of 0.785 for the combined endpoint of unplanned cardiovascular hospitalisation and death [22]. This AUC value is comparable to the predictive performance of NT-proBNP for CHD and CVD outcomes reported in our analysis (C index=0.80–0.83). Furthermore, individuals with a normal NT-proBNP of <125 pg/ml had high negative predictive value of 98% for short-term cardiovascular events [22], suggesting the utility of this biomarker for risk exclusion in individuals with diabetes.

The prognostic utility of NT-proBNP among people with type 2 diabetes has also been investigated in the Dapagliflozin Effect on CardiovascuLAR Events (DECLARE) trial, in which NT-proBNP was measured in 14,565 individuals with type 2 diabetes, of which 33% had elevated levels of NT-proBNP [35]. Inclusion of NT-proBNP improved the prediction of hospitalisation for heart failure/cardiovascular death from 0.76 to 0.82 (*p*<0.001). Similar findings were reported in the Aliskiren in Type 2 Diabetes Using Cardiorenal Endpoints (ALTITUDE) trial, which evaluated 5509 high-risk individuals with type 2 diabetes and CVD and/or CKD. In this study, NT-proBNP alone performed comparably to a base prediction model consisting of 20 clinical variables, achieving a C statistic of 0.723 for a composite cardiovascular outcome, compared with 0.731 with the base model (*p*=0.37). Addition of NT-proBNP to the clinical model significantly enhanced the prediction of composite cardiovascular outcomes (from 0.731 to 0.763, *p*<0.001) as well as the prediction of death (0.744 to 0.779, *p*<0.001) [36]. These findings parallel the incremental predictive value of NT-proBNP that we observed when it was added to the JADE risk model in the HKDB, where the C index improved from 0.73 to 0.75 for the prediction of CHD.

In a recent systematic review of novel biomarkers for predicting cardiovascular events conducted as part of the international consensus report on precision medicine in diabetes [7], a total of 218 studies, covering 195 unique biomarkers, were investigated, and it was concluded in the systematic review that NT-proBNP was one of the most promising biomarkers for prediction of cardiovascular risk in people with type 2 diabetes [3]. In our current analysis, we found that addition of NT-proBNP improved prediction of CV events compared with the JADE risk model, which was developed specifically in a similar population of Chinese individuals with type 2 diabetes, with significant improvement as demonstrated by improvement in ROC, NRI and IDI. This highlights the ability to improve cardiovascular risk stratification in type 2 diabetes by incorporating measurement of NT-proBNP, corroborating the conclusions from the broader literature.

### Prediction of AF

Our analysis also confirmed the utility of NT-proBNP in risk stratification for AF [37]. In the Cardiovascular Health Study including 5445 community-based older adults, NT-proBNP was found to be highly predictive of incident AF, with HR 4.0 (3.2, 5.0) after adjustment for a large number of covariates including age, sex, medication use, blood pressure, echocardiographic parameters, diabetes mellitus and heart failure [38]. In the Framingham Heart Study (FHS), the HR per 1 SD increment in NT-proBNP was 1.73 (95% CI 1.52, 1.96) [39]. In a recent analysis of four prospective cohorts, NT-proBNP was found to be a better predictor of incident AF than newly diagnosed heart failure within the first 2 years of follow-up [40].

### Prediction of renal events

Our analysis identified NT-proBNP as a useful biomarker for incident KF and other renal endpoints among people with type 2 diabetes. In a previous study of 1000 individuals with type 2 diabetes and established CKD (eGFR 20–60 ml/min per 1.73 m^2^ ) and anaemia, elevated NT-proBNP was found to be associated with the risk of incident end stage renal disease, and the association remained prognostically important after adjustment for eGFR, proteinuria and other known predictors of CKD progression [41]. In an examination of 402 candidate biomarkers among a nested case–control cohort of 223 individuals with rapid eGFR decline compared with 258 individuals with stable eGFR course from the PROspective cohort study in patients with type 2 diabetes mellitus for VALIDation of biomarkers (PROVALID study), NT-proBNP, together with kidney injury molecule-1 (KIM-1), were found to be the two best biomarkers for eGFR trajectory during follow-up, with baseline eGFR being an important covariate [16]. Our study expanded these findings, and highlighted the utility of NT-proBNP in predicting hard renal endpoints in type 2 diabetes, including incident cases of KF, as well as a 40% drop in eGFR, which has been proposed as a suitable surrogate endpoint for use in clinical trials of renoprotective agents [42].

### Strengths and limitations

Our study has several strengths, including the detailed documentation of clinical characteristics, and the prospective follow-up of multiple cardiorenal endpoints, which have allowed us to examine the association between baseline NT-proBNP and several clinically relevant endpoints. The high quality of samples in the HKDB has been confirmed in earlier metabolomic studies [43, 44], and all samples were measured on first thaw. We were able to compare the performance of NT-proBNP with clinical risk equations for cardiovascular and renal endpoints that were developed in comparable participant cohorts from the same centre, providing the most appropriate multivariate model for comparison, as highlighted by a recent systematic review on prognostic markers for CVD in type 2 diabetes [3]. We also conducted several sensitivity analyses to establish the robustness of our findings, including using alternative cut-offs of NT-proBNP.

We also acknowledge several limitations of our study. First, we have not included adjustment for all classes of glucose-lowering medications. SGLT2is have been reported to be associated with lowering of NT-proBNP levels, but they were only introduced in Hong Kong in 2014, and hence the percentage of participants on an SGLT2i was likely to be very low at baseline. Furthermore, given that the prescription of SGLT2is was restricted in public hospitals during the period 2014–2019, the number of individuals prescribed an SGLT2i was also limited. In addition, previous studies have suggested that dipeptidyl peptidase-4 (DPP-4) inhibitors have no significant effect on NT-proBNP levels [45].

In summary, in a cohort of Chinese individuals with type 2 diabetes, we showed that NT-proBNP was an independent predictor of cardiovascular and renal endpoints, and improved risk stratification of cardiorenal endpoints beyond that provided by clinical risk equations. The study results suggest that it might be helpful to incorporate NT-proBNP into routine clinical assessment in people with type 2 diabetes for precision prognostics, especially if the cost-effectiveness of the measurements can be demonstrated.

## Supplementary Information

Below is the link to the electronic supplementary material.

ESM (PDF 405 KB)

## Data Availability

The datasets used during the current study are available from the corresponding author (RCWM) upon reasonable request.

## Abbreviations

ACR: Albumin/creatinine ratio
AF: Atrial fibrillation
CHF: Congestive heart failure
C index: Concordance index
CKD: Chronic kidney disease
CKD-EPI: Chronic Kidney Disease Epidemiology Collaboration
DBP: Diastolic blood pressure
HKDB: Hong Kong Diabetes Biobank
HKDR: Hong Kong Diabetes Register
IDI: Integrated discrimination improvement
JADE: Joint Asia Diabetes Evaluation
KF: Kidney failure
NRI: Net reclassification improvement
NT-proBNP: N-terminal pro B-type natriuretic peptide
PVD: Peripheral vascular disease
PWH: Prince of Wales Hospital
RECODe: Risk Equations for Complications Of type 2 Diabetes
ROC: Receiver operating characteristic
SBP: Systolic blood pressure
SGLT2i: Sodium–glucose cotransporter 2 inhibitor
TG: Triacylglycerol
UKPDS: UK Prospective Diabetes Study
WC: Waist circumference

## References

[1] de Boer IH, Khunti K, Sadusky T et al (2022) Diabetes management in chronic kidney disease: a consensus report by the American Diabetes Association (ADA) and Kidney Disease: Improving Global Outcomes (KDIGO). Kidney Int 102(5):974–989. 10.1016/j.kint.2022.08.012 36202661 10.1016/j.kint.2022.08.012

[2] Davies MJ, Aroda VR, Collins BS et al (2022) Management of hyperglycaemia in type 2 diabetes, 2022. A consensus report by the American Diabetes Association (ADA) and the European Association for the Study of Diabetes (EASD). Diabetologia 65(12):1925–1966. 10.1007/s00125-022-05787-2 36151309 10.1007/s00125-022-05787-2 PMC9510507

[3] Ahmad A, Lim LL, Morieri ML et al (2024) Precision prognostics for cardiovascular disease in type 2 diabetes: a systematic review and meta-analysis. Commun Med (Lond) 4(1):11. 10.1038/s43856-023-00429-z 38253823 10.1038/s43856-023-00429-z PMC10803333

[4] Young KG, McInnes EH, Massey RJ et al (2023) Treatment effect heterogeneity following type 2 diabetes treatment with GLP1-receptor agonists and SGLT2-inhibitors: a systematic review. Commun Med (Lond) 3(1):131. 10.1038/s43856-023-00359-w 37794166 10.1038/s43856-023-00359-w PMC10551026

[5] Dennis JM, Young KG, McGovern AP et al (2022) Development of a treatment selection algorithm for SGLT2 and DPP-4 inhibitor therapies in people with type 2 diabetes: a retrospective cohort study. Lancet Digit Health 4(12):e873–e883. 10.1016/S2589-7500(22)00174-1 36427949 10.1016/S2589-7500(22)00174-1

[6] Chung WK, Erion K, Florez JC et al (2020) Precision medicine in diabetes: a consensus report from the American Diabetes Association (ADA) and the European Association for the Study of Diabetes (EASD). Diabetes Care 43(7):1617–1635. 10.2337/dci20-0022 32561617 10.2337/dci20-0022 PMC7305007

[7] Tobias DK, Merino J, Ahmad A et al (2023) Second international consensus report on gaps and opportunities for the clinical translation of precision diabetes medicine. Nat Med 29(10):2438–2457. 10.1038/s41591-023-02502-5 37794253 10.1038/s41591-023-02502-5 PMC10735053

[8] Stevens RJ, Kothari V, Adler AI, Stratton IM, United Kingdom Prospective Diabetes Study (UKPDS) Group (2001) The UKPDS risk engine: a model for the risk of coronary heart disease in type II diabetes (UKPDS 56). Clin Sci (Lond) 101(6):671–679. 10.1042/cs1010671 11724655

[9] Khera AV, Chaffin M, Aragam KG et al (2018) Genome-wide polygenic scores for common diseases identify individuals with risk equivalent to monogenic mutations. Nat Genet 50(9):1219–1224. 10.1038/s41588-018-0183-z 30104762 10.1038/s41588-018-0183-z PMC6128408

[10] Tam CHT, Lim CKP, Luk AOY et al (2021) Development of genome-wide polygenic risk scores for lipid traits and clinical applications for dyslipidemia, subclinical atherosclerosis, and diabetes cardiovascular complications among East Asians. Genome Med 13(1):29. 10.1186/s13073-021-00831-z 33608049 10.1186/s13073-021-00831-z PMC7893928

[11] Morieri ML, Gao H, Pigeyre M et al (2018) Genetic tools for coronary risk assessment in type 2 diabetes: a cohort study from the ACCORD clinical trial. Diabetes Care 41(11):2404–2413. 10.2337/dc18-0709 30262460 10.2337/dc18-0709 PMC6196830

[12] Jin Q, Luk AO, Lau ESH et al (2022) Nonalbuminuric diabetic kidney disease and risk of all-cause mortality and cardiovascular and kidney outcomes in type 2 diabetes: findings from the Hong Kong Diabetes Biobank. Am J Kidney Dis 80(2):196-206 e191. 10.1053/j.ajkd.2021.11.011 34999159 10.1053/j.ajkd.2021.11.011

[13] Mueller C, McDonald K, de Boer RA et al (2019) Heart Failure Association of the European Society of Cardiology practical guidance on the use of natriuretic peptide concentrations. Eur J Heart Fail 21(6):715–731. 10.1002/ejhf.1494 31222929 10.1002/ejhf.1494

[14] Tsutsui H, Albert NM, Coats AJS et al (2023) Natriuretic peptides: role in the diagnosis and management of heart failure: a scientific statement from the heart failure association of the European society of cardiology, heart failure society of America and Japanese Heart Failure Society. J Card Fail 29(5):787–804. 10.1016/j.cardfail.2023.02.009 37117140 10.1016/j.cardfail.2023.02.009

[15] Ibrahim NE, Burnett JC Jr, Butler J et al (2020) Natriuretic peptides as inclusion criteria in clinical trials: a JACC: heart failure position paper. JACC Heart Fail 8(5):347–358. 10.1016/j.jchf.2019.12.010 32171762 10.1016/j.jchf.2019.12.010

[16] Kammer M, Heinzel A, Willency JA et al (2019) Integrative analysis of prognostic biomarkers derived from multiomics panels helps discrimination of chronic kidney disease trajectories in people with type 2 diabetes. Kidney Int 96(6):1381–1388. 10.1016/j.kint.2019.07.025 31679767 10.1016/j.kint.2019.07.025

[17] Chan JCN, Lim LL, Luk AOY et al (2019) From Hong Kong diabetes register to JADE program to RAMP-DM for data-driven actions. Diabetes care 42(11):2022–2031. 10.2337/dci19-0003 31530658 10.2337/dci19-0003

[18] Jiang G, Luk AO, Tam CHT et al (2020) Obesity, clinical, and genetic predictors for glycemic progression in Chinese patients with type 2 diabetes: a cohort study using the Hong Kong Diabetes Register and Hong Kong Diabetes Biobank. PLoS Med 17(7):e1003209. 10.1371/journal.pmed.1003209 32722720 10.1371/journal.pmed.1003209 PMC7386560

[19] Jiang G, Luk AO, Tam CHT et al (2022) Clinical predictors and long-term impact of acute kidney injury on progression of diabetic kidney disease in Chinese patients with type 2 diabetes. Diabetes 71(3):520–529. 10.2337/db21-0694 35043149 10.2337/db21-0694 PMC8893937

[20] Piwernetz K, Home PD, Snorgaard O, Antsiferov M, Staehr-Johansen K, Krans M (1993) Monitoring the targets of the St Vincent Declaration and the implementation of quality management in diabetes care: the DIABCARE initiative. The DIABCARE Monitoring Group of the St Vincent Declaration Steering Committee. Diabet Med 10(4):371–377. 10.1111/j.1464-5491.1993.tb00083.x 8508624 10.1111/j.1464-5491.1993.tb00083.x

[21] Levey AS, Stevens LA, Schmid CH et al (2009) A new equation to estimate glomerular filtration rate. Ann Intern Med 150(9):604–612. 10.7326/0003-4819-150-9-200905050-00006 19414839 10.7326/0003-4819-150-9-200905050-00006 PMC2763564

[22] Huelsmann M, Neuhold S, Strunk G et al (2008) NT-proBNP has a high negative predictive value to rule-out short-term cardiovascular events in patients with diabetes mellitus. Eur Heart J 29(18):2259–2264. 10.1093/eurheartj/ehn334 18650200 10.1093/eurheartj/ehn334

[23] McDonagh TA, Metra M, Adamo M et al (2021) 2021 ESC Guidelines for the diagnosis and treatment of acute and chronic heart failure. Eur Heart J 42(36):3599–3726. 10.1093/eurheartj/ehab368 34447992 10.1093/eurheartj/ehab368

[24] Aoki S, Yamagishi K, Kihara T et al (2023) Risk factors for pre-heart failure or symptomatic heart failure based on NT-proBNP. ESC Heart Fail 10(1):90–99. 10.1002/ehf2.14149 36151844 10.1002/ehf2.14149 PMC9871651

[25] Yang X, Kong AP, So WY et al (2006) Effects of chronic hyperglycaemia on incident stroke in Hong Kong Chinese patients with type 2 diabetes. Diabetes Metab Res Rev 23(3):220–226. 10.1002/dmrr.675 10.1002/dmrr.675 16871645

[26] Yang X, So WY, Kong AP et al (2008) Development and validation of a total coronary heart disease risk score in type 2 diabetes mellitus. Am J Cardiol 101(5):596–601. 10.1016/j.amjcard.2007.10.019 18308005 10.1016/j.amjcard.2007.10.019

[27] Yang XL, So WY, Kong AP et al (2006) End-stage renal disease risk equations for Hong Kong Chinese patients with type 2 diabetes: Hong Kong Diabetes Registry. Diabetologia 49(10):2299–2308. 10.1007/s00125-006-0376-3 16944095 10.1007/s00125-006-0376-3

[28] Yang XL, So WY, Kong AP et al (2007) Modified end-stage renal disease risk score for Chinese type 2 diabetic patients—the Hong Kong Diabetes Registry. Diabetologia 50(6):1348–1350. 10.1007/s00125-007-0639-7 17431580 10.1007/s00125-007-0639-7

[29] Hayes AJ, Leal J, Gray AM, Holman RR, Clarke PM (2013) UKPDS outcomes model 2: a new version of a model to simulate lifetime health outcomes of patients with type 2 diabetes mellitus using data from the 30 year United Kingdom Prospective Diabetes Study: UKPDS 82. Diabetologia 56(9):1925–1933. 10.1007/s00125-013-2940-y 23793713 10.1007/s00125-013-2940-y

[30] Basu S, Sussman JB, Berkowitz SA et al (2018) Validation of Risk Equations for Complications of Type 2 Diabetes (RECODe) using individual participant data from diverse longitudinal cohorts in the U.S. Diabetes Care 41(3):586–595. 10.2337/dc17-2002 29269511 10.2337/dc17-2002 PMC5829967

[31] McDonagh TA, Metra M, Adamo M et al (2023) 2023 Focused update of the 2021 ESC Guidelines for the diagnosis and treatment of acute and chronic heart failure. Eur Heart J 44(37):3627–3639. 10.1093/eurheartj/ehad195 37622666 10.1093/eurheartj/ehad195

[32] Heidenreich PA, Bozkurt B, Aguilar D et al (2022) 2022 AHA/ACC/HFSA guideline for the management of heart failure: executive summary: a report of the American College of Cardiology/American Heart Association Joint Committee on Clinical Practice Guidelines. J Am Coll Cardiol 79(17):1757–1780. 10.1016/j.jacc.2021.12.011 35379504 10.1016/j.jacc.2021.12.011

[33] Januzzi JL Jr, Xu J, Li J et al (2020) Effects of canagliflozin on amino-terminal pro-B-type natriuretic peptide: implications for cardiovascular risk reduction. J Am Coll Cardiol 76(18):2076–2085. 10.1016/j.jacc.2020.09.004 33121714 10.1016/j.jacc.2020.09.004

[34] Natriuretic Peptides Studies Collaboration, Willeit P, Kaptoge S et al (2016) Natriuretic peptides and integrated risk assessment for cardiovascular disease: an individual-participant-data meta-analysis. Lancet Diabetes Endocrinol 4(10):840–849. 10.1016/S2213-8587(16)30196-6 27599814 10.1016/S2213-8587(16)30196-6 PMC5035346

[35] Zelniker TA, Morrow DA, Mosenzon O et al (2021) Relationship between baseline cardiac biomarkers and cardiovascular death or hospitalization for heart failure with and without sodium-glucose co-transporter 2 inhibitor therapy in DECLARE-TIMI 58. Eur J Heart Fail 23(6):1026–1036. 10.1002/ejhf.2073 33269486 10.1002/ejhf.2073

[36] Malachias MVB, Jhund PS, Claggett BL et al (2020) NT-proBNP by itself predicts death and cardiovascular events in high-risk patients with type 2 diabetes mellitus. J Am Heart Assoc 9(19):e017462. 10.1161/JAHA.120.017462 32964800 10.1161/JAHA.120.017462 PMC7792415

[37] Chang KW, Hsu JC, Toomu A, Fox S, Maisel AS (2017) Clinical applications of biomarkers in atrial fibrillation. Am J Med 130(12):1351–1357. 10.1016/j.amjmed.2017.08.003 28822701 10.1016/j.amjmed.2017.08.003

[38] Patton KK, Ellinor PT, Heckbert SR et al (2009) N-terminal pro-B-type natriuretic peptide is a major predictor of the development of atrial fibrillation: the Cardiovascular Health Study. Circulation 120(18):1768–1774. 10.1161/CIRCULATIONAHA.109.873265 19841297 10.1161/CIRCULATIONAHA.109.873265 PMC4132053

[39] Staerk L, Preis SR, Lin H et al (2020) Protein biomarkers and risk of atrial fibrillation: the FHS. Circ Arrhythm Electrophysiol 13(2):e007607. 10.1161/CIRCEP.119.007607 31941368 10.1161/CIRCEP.119.007607 PMC7031024

[40] Werhahn SM, Becker C, Mende M et al (2022) NT-proBNP as a marker for atrial fibrillation and heart failure in four observational outpatient trials. ESC Heart Fail 9(1):100–109. 10.1002/ehf2.13703 34850596 10.1002/ehf2.13703 PMC8788004

[41] Desai AS, Toto R, Jarolim P et al (2011) Association between cardiac biomarkers and the development of ESRD in patients with type 2 diabetes mellitus, anemia, and CKD. Am J Kidney Dis 58(5):717–728. 10.1053/j.ajkd.2011.05.020 21820220 10.1053/j.ajkd.2011.05.020

[42] Levey AS, Gansevoort RT, Coresh J et al (2020) Change in albuminuria and GFR as end points for clinical trials in early stages of CKD: a scientific workshop sponsored by the national kidney foundation in collaboration with the US Food and Drug Administration and European Medicines Agency. Am J Kidney Dis 75(1):84–104. 10.1053/j.ajkd.2019.06.009 31473020 10.1053/j.ajkd.2019.06.009

[43] Jin Q, Lau ESH, Luk AO et al (2022) High-density lipoprotein subclasses and cardiovascular disease and mortality in type 2 diabetes: analysis from the Hong Kong Diabetes Biobank. Cardiovasc Diabetol 21(1):293. 10.1186/s12933-022-01726-y 36587202 10.1186/s12933-022-01726-y PMC9805680

[44] Jin Q, Lau ESH, Luk AO et al (2024) Circulating metabolomic markers linking diabetic kidney disease and incident cardiovascular disease in type 2 diabetes: analyses from the Hong Kong Diabetes Biobank. Diabetologia 67(5):837–849. 10.1007/s00125-024-06108-5 38413437 10.1007/s00125-024-06108-5 PMC10954952

[45] Fadini GP, Bonora BM, Albiero M, Zaninotto M, Plebani M, Avogaro A (2017) DPP-4 inhibition has no acute effect on BNP and its N-terminal pro-hormone measured by commercial immune-assays. A randomized cross-over trial in patients with type 2 diabetes. Cardiovasc Diabetol 16(1):22. 10.1186/s12933-017-0507-9 28183314 10.1186/s12933-017-0507-9 PMC5301429

[46] Yang X, Ma RC, So WY et al (2008) Development and validation of a risk score for hospitalization for heart failure in patients with type 2 diabetes mellitus. Cardiovasc Diabetol 7:9. 10.1186/1475-2840-7-9 18430204 10.1186/1475-2840-7-9 PMC2377240

