# Supplementary material for: NT-proBNP improves prediction of cardiorenal complications in type 2 diabetes: the Hong Kong Diabetes Biobank
Source: Diabetologia. 2024 Nov 7;68(2):342–56. doi: 10.1007/s00125-024-06299-x (PMC11732878; doi:10.1007/s00125-024-06299-x)
Supplement: Supplementary file 1 — ESM (PDF 405 KB) [file 125_2024_6299_MOESM1_ESM.pdf]

SUPPLEMENTARY MATERIAL:

Hong Kong Diabetes Biobank Study Group Members

Ronald C.W. Ma [1,2,3,4], Juliana C.N. Chan [1,2,3,4], Risa Ozaki[1,2], Andrea O.Y. Luk[1,2,3,4], Wing-yee So[1,2], Ka-fai Lee[5], Shing-chung Siu[6], Grace Hui[6], Chiu-chi Tsang[7], Kam-piu Lau[8], Jenny Y.Y. Leung[9], Man-wo Tsang[10], Grace Kam[10], Elaine Cheung[10], Ip-tim Lau[11], June K.Y. Li[12], Vincent T.F. Yeung[13], Jo Jo Kwan[13], Samuel Fung[14], Stanley Lo[15], Emmy Lau[15], Yuk-lun Cheng[16], Stephen K.W. Tsui[17], Yu Huang[17], Huiyao Lan[1,3], Weichuan Yu[18], Brian Tomlinson[1], Si Lok[19], Ting-fung Chan[20], Kevin Y.L. Yip[21], Cheuk-chun Szeto[1,3], Xiaodan Fan[22], Nelson L.S. Tang[3, 23], Xiaoyu Tian[17], Claudia H.T. Tam[1,2,4], Guozhi Jiang[1,2,4], Shi Mai[17], Baoqi Fan[1,2,4], Fei Xie[1], Sen Zhang[18], Pu Yu[18], Meng Wang[18], Heung-man Lee[1], Cadmon K.P. Lim [1,2,4], Fangying Xie[1], Alex C.W. Ng[1], Grace P.Y. Cheung[1], Alice P.S. Kong[1,2], Elaine Y.K. Chow[1,2], Ming-wai Yeung[1], Chun-chung Chow[1], Kitty K.T. Cheung[1], Rebecca Y.M. Wong[1], Hon-cheong So[17], Katie K.H. Chan[1,2], Chin-san Law[11], Anthea K.Y. Lock[11], Ingrid K.Y. Tsang[11], Susanna C.P. Chan[11], Yin-wah Chan[11], Cherry Chiu[2], Chi-sang Hung[10], Cheuk-wah Ho[10], Ivy H.Y. Ng[10], Maria W.H. Mak[7], Kai-man Lee[7], Candy H.S. Leung[13], Ka-wah Lee[12], Hui-ming Chan[12], Winnie Wat[15], Tracy Lau[15], Cheuk-yiu Law[14], Ryan H.Y. Chan[14], Candice Lau[1], Pearl Tsang[1], Vince Chan[1], Lap-ying Ho[1], Eva Wong[1], Josephine Chan[1], Sau-fung Lam[1], Jessy Pang[1], Yee-mui Lee[1]

[1] Department of Medicine and Therapeutics, The Chinese University of Hong Kong, Hong Kong

[2] Hong Kong Institute of Diabetes and Obesity, The Chinese University of Hong Kong, Hong Kong

[3] Li Ka Shing Institute of Health Sciences, The Chinese University of Hong Kong, Hong Kong

[4] Chinese University of Hong Kong-Shanghai Jiao Tong University Joint Research Centre in Diabetes Genomics and Precision Medicine

[5] Department of Medicine and Geriatrics, Kwong Wah Hospital, Hong Kong

[6] Diabetes Centre, Tung Wah Eastern Hospital, Hong Kong

[7] Diabetes and Education Centre, Alice Ho Miu Ling Nethersole Hospital, Hong Kong

[8] North District Hospital, Hong Kong

[9] Department of Medicine and Geriatrics, Ruttonjee Hospital, Hong Kong

[10] Department of Medicine and Geriatrics, United Christian Hospital, Hong Kong

[11] Tseung Kwan O Hospital, Hong Kong

[12] Department of Medicine, Yan Chai Hospital, Hong Kong

[13] Centre for Diabetes Education and Management, Our Lady of Maryknoll Hospital, Hong Kong

[14] Department of Medicine and Geriatrics, Princess Margaret Hospital, Hong Kong

[15] Department of Medicine, Pamela Youde Nethersole Eastern Hospital, Hong Kong

[16] Department of Medicine, Alice Ho Miu Ling Nethersole Hospital, Hong Kong

[17] School of Biomedical Sciences, The Chinese University of Hong Kong

[18] Department of Electronic and Computer Engineering, Hong Kong University of Science and Technology, Hong Kong

[19] The Centre for Applied Genomics, The Hospital for Sick Children, Toronto, Canada

[20] School of Life Sciences, The Chinese University of Hong Kong, Hong Kong

[21] Department of Computer Science and Engineering, The Chinese University of Hong Kong, Hong Kong

[22] Department of Statistics, The Chinese University of Hong Kong, Hong Kong

[23] Department of Chemical Pathology, The Chinese University of Hong Kong, Hong Kong

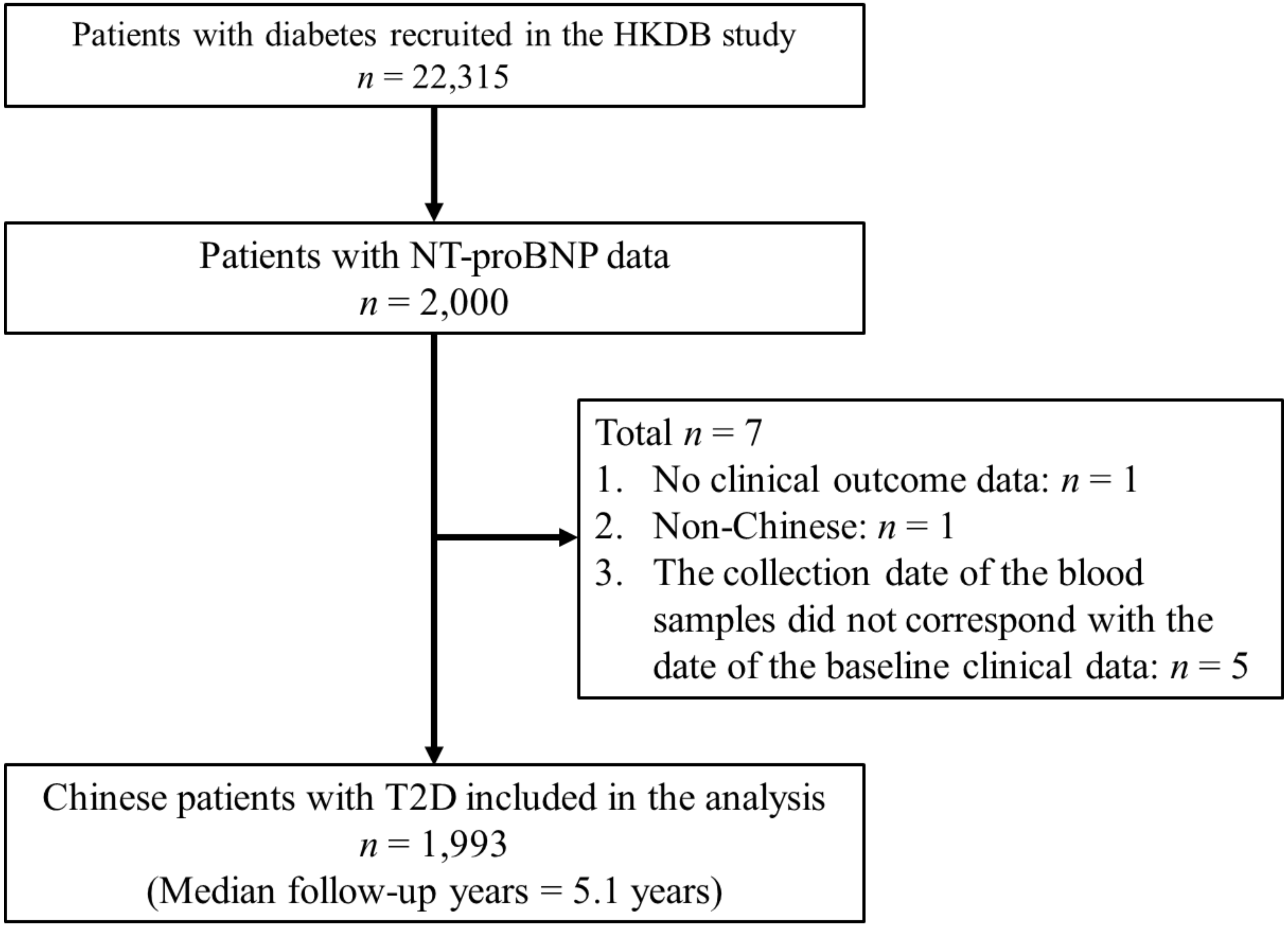

ESM Fig. 1. Number of subjects included in the analyses.

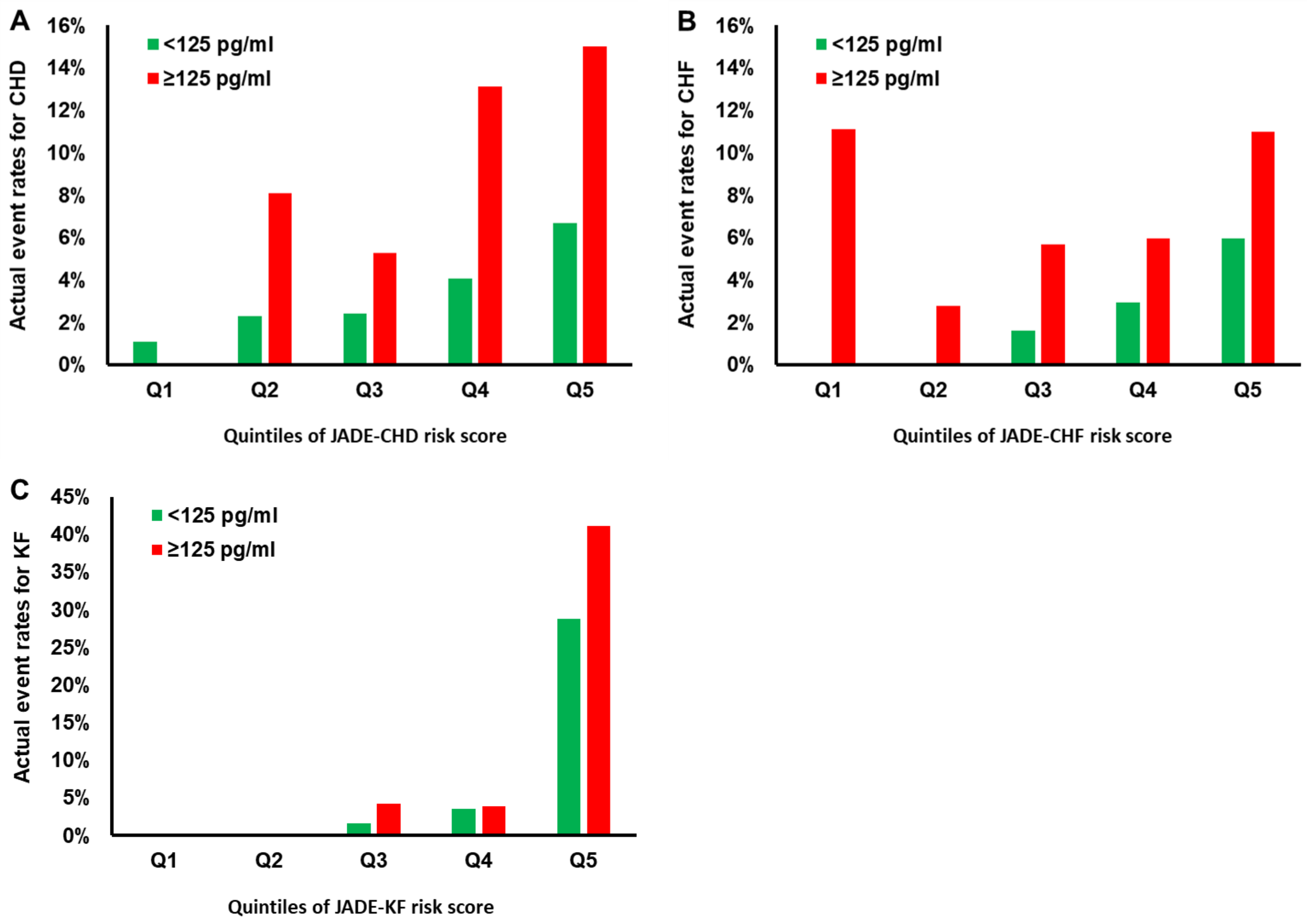

ESM Fig. 2. Actual event rates for incident cardio-renal outcomes, stratified by the quintiles of the corresponding JADE risk score and NT-proBNP. A) CHD, coronary heart disease; B) CHF, congestive heart failure; C) KF, kidney failure.

ESM Table 1. Comparison of the baseline clinical characteristics between the participants who were included and those who were not included in the current study from the HKDB cohort.

Characteristic	Included in the study (N = 1993)	Not included in the study (N = 18429)
Clinical characteristics at baseline		
Male	59.8 (1192)	58.4 (10772)
Age (years)	61.1 ± 11.0	61.2 ± 11.3
Age at onset (years)	49.7 ± 11.6	50.0 ± 11.5
Duration of diabetes (years)	11.3 ± 8.66	11.2 ± 8.89
Smoking status		
Non-smoker	66.1 (1317)	65.9 (12135)
Ex-smoker	22.0 (438)	21.1 (3883)
Current smoker	11.9 (237)	13.0 (2393)
Body height (m)	1.62 ± 0.08	1.62 ± 0.09
Body weight (kg)	68.4 (60.0–78.0)	68.1 (59.7 - 78.0)
BMI (kg/m^2)	25.9 (23.4–29.1)	25.8 (23.3 - 28.8)
WC (cm)		
Men	93.9 ± 11.3	94.3 ± 11.3
Women	89.5 ± 11.4	89.0 ± 11.4
Hip circumference (cm)	94.7 ± 8.5	97.5 ± 8.82
WHR	0.97 ± 0.08	0.94 ± 0.07
HbA_{1c} (mmol/mol)	55.0 (49.0–65.0)	
HbA_{1c} (%)	7.20 (6.60–8.10)	7.30 (6.60 - 8.30)
Total cholesterol (mmol/l)	4.26 (3.73–4.86)	4.18 (3.62 - 4.80)
TGs (mmol/l)	1.34 (0.96–2.00)	1.35 (0.96 - 1.96)
HDL-cholesterol (mmol/l)	1.22 (1.01–1.47)	1.17 (0.99 - 1.40)
LDL-cholesterol (mmol/l)	2.26 (1.81–2.75)	2.23 (1.78 - 2.75)
SBP (mmHg)	135 ± 18.4	135 ± 17.9
DBP (mmHg)	74.0 ± 11.4	74.9 ± 11.4
ACR	3.90 (1.10–24.6)	2.80 (1.00 - 12.5)
eGFR (min/ml per 1.73 m^2)	75.9 ± 26.3	78.6 ± 24.1
Treatment at baseline		
Lipid-lowering drug	68.7 (1359)	69.3 (12656)
Blood pressure anti-hypertensive	77.2 (1523)	72.1 (13171)
Oral glucose-lowering drug	86.0 (1682)	90.2 (12939)
Insulin treatment	38.1 (749)	31.8 (5779)
History of cardiorenal complication at baseline		
AF	2.6 (52)	2.8 (506)
CHD	17.3 (345)	17.4 (3209)
Stroke	8.8 (176)	8.8 (1617)
PVD	1.6 (32)	1.2 (230)
CVDs	24.9 (496)	24.9 (4591)
CHF	5.0 (100)	3.9 (716)
CKD	32.9 (655)	26.0 (4798)
KF	1.9 (37)	1.9 (358)

40% drop in eGFR	9.1 (181)	7.6 (1380)
Composite renal endpoint	9.3 (186)	7.8 (1405)

Data were expressed as percentage (n), mean ± SD or median (Q1-Q3). We have further excluded patients who are either non-Chinese, non-T2D (e.g. those with type 1 diabetes) or lacking clinical outcome data from the analysis.

ESM Table 2. Association of NT-proBNP with incident diabetes cardio-renal complications (sensitivity analysis).

Outcome	*n*		log-transformed NT pro-BNP		Top quintile of NT pro-BNP vs. other		Binary NT pro-BNP ((≥400 vs. <400)	
	**Event**	**Non-event**	**HR (95% CI)**	***P***	**HR (95% CI)**	***P***	**HR (95% CI)**	***P***
Atrial fibrillation	49	1,852	1.84 (1.56 - 2.17)	8.5×10^{-13}	4.82 (2.58 - 9.01)	7.8×10^{-7}	6.81 (3.69 - 12.6)	8.5×10^{-10}
Coronary heart disease	65	1,572	1.75 (1.50 - 2.05)	1.8×10^{-12}	5.11 (2.98 - 8.76)	3.0×10^{-9}	3.94 (2.09 - 7.45)	2.4×10^{-5}
Cardiovascular disease	111	1,376	1.73 (1.53 - 1.95)	3.6×10^{-18}	4.38 (2.90 - 6.62)	2.4×10^{-12}	4.42 (2.70 - 7.23)	3.2×10^{-9}
Congestive heart failure	49	1,835	1.93 (1.62 - 2.29)	8.7×10^{-14}	5.49 (2.88 - 10.5)	2.2×10^{-7}	6.39 (3.50 - 11.7)	1.7×10^{-9}
Chronic kidney disease	168	1,163	1.58 (1.39 - 1.79)	2.5×10^{-12}	2.47 (1.69 - 3.61)	3.0×10^{-6}	2.87 (1.71 - 4.83)	6.8×10^{-5}
Kidney failure	144	1,802	2.02 (1.80 - 2.27)	2.4×10^{-32}	4.86 (3.39 - 6.99)	1.1×10^{-17}	4.56 (3.12 - 6.68)	5.7×10^{-15}
40% drop in eGFR	426	1,373	1.66 (1.54 - 1.79)	1.7×10^{-40}	2.85 (2.30 - 3.54)	2.9×10^{-21}	3.46 (2.70 - 4.45)	2.8×10^{-22}
Composite renal endpoint	422	1,372	1.64 (1.52 - 1.77)	2.8×10^{-38}	2.77 (2.23 - 3.45)	6.3×10^{-20}	3.29 (2.55 - 4.25)	5.2×10^{-20}

HRs and 95% CIs were reported according to top quintile of NT-proBNP or high NT-proBNP levels (i.e. high NT-proBNP was defined as ≥400 pg/ml).

P was obtained from Cox regression model with the adjustments of sex, age and duration of diabetes.

ESM Table 3. Number of subjects and actual event rates for incident cardio-renal outcomes, stratified by the quintiles of the corresponding JADE risk score and NT-proBNP.

Outcome	Quintiles of JADE risk score	NT-proBNP groups					
		<125 pg/ml			≥125 pg/ml		
		**Control *n***	**Case *n***	**Case %**	**Control *n***	**Case *n***	**Case %**
Coronary heart disease	Q1	274	3	1.1%	20	0	0.0%
	Q2	296	7	2.3%	34	3	8.1%
	Q3	201	5	2.4%	54	3	5.3%
	Q4	165	7	4.1%	53	8	13.1%
	Q5	112	8	6.7%	102	18	15.0%
Congestive heart failure	Q1	317	0	0.0%	8	1	11.1%
	Q2	310	0	0.0%	35	1	2.8%
	Q3	243	4	1.6%	50	3	5.7%
	Q4	166	5	2.9%	79	5	6.0%
	Q5	95	6	5.9%	162	20	11.0%
Kidney failure	Q1	355	1	0.3%	19	0	0.0%
	Q2	344	0	0.0%	44	0	0.0%
	Q3	189	3	1.6%	68	3	4.2%
	Q4	135	5	3.6%	75	3	3.8%
	Q5	94	38	28.8%	113	79	41.1%

ESM Table 4. NRI and IDI of NT-proBNP in predicting incident cardio-renal complications, over the UKPDS 82 and RECODe risk equations.

**Outcome**	**Clinical risk score [Ref]**	**Predictors included in the clinical risk score**	***n*** **Case**	**Control**	**Net reclassification improvement index** Continuous NRI (95% CI)	P_{NRI}	**Integrated discrimination index** IDI (95% CI)	P_{IDI}	**Relative integrated discrimination index** rIDI (95% CI)	P_{IDI}
CHD	UKPDS 82 risk equation for ischemic heart disease [1]	Sex, age at diagnosis of diabetes, SBP, HDL cholesterol, LDL cholesterol, eGFR, history of amputation, the presence of PVD, history of CHF	63	1,545	71.6% (45.8% - 97.3%)	<0.05	0.026 (0.010 - 0.052)	$<1.0\times10^{-3}$	1.153 (0.266 - 2.040)	<0.05
	RECODe risk equation for myocardial infarction [2]	sex, age, current smoking status, SBP, history of CVD, blood pressure-lowering drugs, statins, anticoagulants, HbA_{1c}, total cholesterol, HDL cholesterol, serum creatinine, urinary ACR	62	1,292	72.0% (45.4% - 97.6%)	<0.05	0.015 (0.004 - 0.038)	1.0×10^{-3}	0.153 (-0.167 - 0.473)	>0.05

NT-proBNP was used as a binary variable with a predefined threshold of 125pg/ml (i.e. high NT-proBNP was defined as ≥125pg/ml). CHD, coronary heart disease; CHF, congestive heart failure; KF, kidney failure; BMI, body mass index; ACR, albumin-creatinine ratio; eGFR, estimated glomerular filtration rate. For the NRI and IDI analysis, old model includes the clinical risk score. New model includes old model and NT-proBNP. P_{NRI} was obtained from the test with the null hypothesis that "Continuous NRI is equal to zero". P_{IDI} was obtained from the test with the null hypothesis that "Continuous IDI is equal to zero". P_{rIDI} was obtained from the test with the null hypothesis that "Relative IDI is equal to zero".

References:

[1] Hayes AJ, Leal J, Gray AM et al. UKPDS outcomes model 2: a new version of a model to simulate lifetime health outcomes of patients with type 2 diabetes mellitus using data from the 30 year United Kingdom Prospective Diabetes Study: UKPDS 82. *Diabetologia. 2013 Sep;56(9):1925-33*.

[2] Basu S, Sussman JB, Berkowitz SA et al. Validation of Risk Equations for Complications of Type 2 Diabetes (RECODe) Using Individual Participant Data From Diverse Longitudinal Cohorts in the U.S. *Diabetes Care. 2018 Mar;41(3):586-595*.
